# Characteristics of and Factors Associated With Partner Service Uptake Cascade Among People With Newly Reported HIV/AIDS Diagnoses in Southeastern China in 2022: Cross-Sectional Survey

**DOI:** 10.2196/59095

**Published:** 2024-09-09

**Authors:** Jun Jiang, Lin Chen, Wei Cheng, Wanjun Chen, Jiezhe Yang, Yun Xu, Xin Zhou, Xiaohong Pan, Chengliang Chai

**Affiliations:** 1 Zhejiang Provincial Center for Disease and Control and Prevention Hangzhou China

**Keywords:** HIV/AIDS, partner service, testing, nonmarital partnership, heterosexual, homosexual, China

## Abstract

**Background:**

HIV notification and testing integrated into partner service (PS) practices among HIV-positive individuals have been proven to be an efficient approach for case finding, although it remains a weak link in China. Although nonmarital sexual activities accounted for a large proportion of newly diagnosed HIV-positive cases in China, little is known about PS uptake and associated factors within nonmarital partnerships.

**Objective:**

This study aimed to describe HIV PS utilization and its associated factors among HIV-positive individuals with nonmarital sexual partners.

**Methods:**

We recruited newly diagnosed HIV-positive individuals who had nonmarital sexual partners in 2022 in Zhejiang Province and offered them PS. We described the PS uptake cascade within sexual partner categories and analyzed the associated factors with 3 primary outcomes from the participants’ perspective: nonmarital partner enumeration, HIV testing, and HIV positivity.

**Results:**

In this study, 3509 HIV-positive individuals were recruited as participants, and they enumerated 2507 nonmarital sex partners (2507/14,556, 17.2% of all nonmarital sex partners) with contact information. Among these, 43.1% (1090/2507) underwent an HIV test, with an HIV-positive rate of 28.3% (309/1090). Heterosexual commercial partners were the least likely of being enumerated (441/4292, 10.3%) and had the highest HIV-positive rate (40/107, 37.4%). At the participant level, 48.1% (1688/3509) of the participants enumerated at least one nonmarital sex partner with contact information, 52.7% (890/1688) had a sex partner tested for HIV, and 31% (276/890) had at least one nonmarital sex partner who tested positive. Multivariate analysis indicated that gender and transmission route were associated with both nonmarital sex partner enumeration and HIV testing. Age and occupation were associated with nonmarital sex partner enumeration and HIV positivity. Compared with participants who had no regular nonmarital sex partner, those who had a regular nonmarital sex partner were more likely to enumerate nonmarital sex partners (adjusted odds ratio [aOR] 3.017, 95% CI 2.560-3.554), have them get tested for HIV (aOR 1.725, 95% CI 1.403-2.122), and have an HIV-positive nonmarital sex partner (aOR 1.962, 95% CI 1.454-2.647).

**Conclusions:**

The percentage of partner enumeration was low, and HIV testing rate was moderate among nonmarital partnerships of HIV-positive individuals. More efforts should be made to improve PS practices among HIV-positive individuals and address the gap in partner enumeration, especially for heterosexual commercial nonmarital partnerships. Additionally, enhancing PS operational skills among health care personnel could increase the overall efficiency of PS uptake in China.

## Introduction

Promptly detecting HIV-positive individuals is crucial for identifying more infections and providing further care and treatment [1]. After reaching a peak in 2019, the number of newly diagnosed HIV/AIDS cases in China decreased in 2020-2022, while 38% were diagnosed late [2]. By the end of 2020, the progress toward the Joint United Nations Programme on HIV/AIDS (UNAIDS) 95-95-95 (95% of people who are living with HIV to know their HIV status, 95% of people who know that they are living with HIV to be on lifesaving antiretroviral treatment, and 95% of people who are on treatment to be virally suppressed) global target [3] was 79%, 93%, and 96% [4], respectively, in China, with over 20% of HIV-positive individuals still unaware of their serostatus [5], a large gap to reaching the first 95-95-95 goal of the UNAIDS target. These statistics contribute to the high risk for HIV transmission [6] and pose a great challenge in curbing the epidemic.

Many modalities or strategies have been implemented to expand HIV testing and improve the efficiency of HIV case-finding, including promoting couples’ voluntary HIV counseling and testing [7], providing free HIV self-testing kits for men who have sex with men (MSM) and their sexual partners [8], contact tracing, and molecular epidemiologic investigation [9,10]. The European Centre for Disease Prevention and Control and World Health Organization (WHO) have strongly recommended that voluntary assisted partner notification services should be offered as part of a comprehensive package of testing and care offered to people with HIV [11,12], and this is a critical strategy for targeted HIV case identification [13].

Like the HIV antiretroviral therapy cascade, HIV partner services (PS) consist of several stages, including partner enumeration and notification, partner testing, and referring HIV-positive partners to care and treatment. The population at risk of HIV infection can only be identified once each step of the PS cascade has been fully accomplished. To increase the uptake of partner notification and testing services, many studies have been conducted to explore the acceptability of innovative geosocial networking applications [14] and testing initiated by participants or health professionals [15], assisted partner notifications, or PS [16,17].

In the United States, among index-partner pairs with notifiable partners, 97.2% (41,762/42,973) notified their partner, and 52.3% (21,842/41,762) of notified partners were tested for HIV [18]. Among HIV-positive female index clients in Kenya, 86% of their male partners were reached about exposure notification and HIV test services [16]. Partner notification programs for sexually transmitted diseases (STD) or HIV are feasible in China [19], although the notification and test rates are moderate [20,21]. The pooled uptake rate of HIV testing for couples among Chinese people diagnosed with HIV was 65% [21]. Among HIV-positive MSM who were married and diagnosed in 2014, 69.2% (3517/5081) had their wives tested for HIV within 180 days after HIV diagnosis [22]. A cross-sectional study conducted among HIV-infected MSM attending HIV care clinics in Guangzhou resulted in a partner notification rate of 63.6% (117/184), including 66.2% (100/151) among regular partners and 20% (20/100) among casual partners [23]. A randomized controlled trial conducted among newly diagnosed MSM in northern China found that 35% of participants in the assisted partner notification group (provided with HIV self-testing kits for sexual partners to take tests at home) had any sexual partner accessing HIV testing compared with 17% in the passive partner notification group [24].

In China, the primary route of HIV infection has shifted from blood transmission to sexual transmission [2,4]. According to the Regulation on AIDS Prevention and Control of China, HIV-infected individuals are obligated to notify their sexual partners of their HIV status [25]. The Yunnan Provincial AIDS Prevention and Control Regulation specifies that HIV-infected individuals should promptly inform their spouses or sexual partners of the fact that they are infected with HIV. Trained staff in medical institutions have the right to inform spouses or sexual partners if HIV-infected individuals refuse to notify them [26]. For sexual partners who are not in a marital relationship, HIV notification and testing are not compulsory, and they are not bound by the marital relationship. Although the number of individuals infected from nonmarital relationships, including commercial heterosexual contact, nonmarital noncommercial heterosexual contact, and MSM, accounted for a large proportion of newly diagnosed HIV-positive individuals in China [27,28], research on PS among nonmarital partnerships is limited.

Studies have explored the factors and barriers associated with PS from different perspectives, including demographic characteristics [18], concerns about relationship repercussions, trust or violence at the relationship level [29-31], facilities, human resources and feasibility from the facilitator and logistic prospectives [32], and stigma and discrimination at the social norm level. This study aimed to describe the utilization of PS among HIV-infected individuals in nonmarital partnerships and explore the factors associated with enumeration, testing, and HIV positivity of nonmarital partners in eastern China, specifically in Zhejiang province

## Methods

### Ethical Considerations

The study was approved by the ethical review board of Zhejiang Provincial Center for Disease Control and Prevention (CDC; IRB approval number: 2019-039). Informed consent information was provided to participants during the HIV notification procedure. No risk was involved with participating in the study, the confidentiality of participants was properly protected, and all data were deidentified before analyzing. All study procedures were conducted under the approved guidelines and regulations.

### Study Design and Participants

This cross-sectional study was designed to describe the utilization of PS among newly diagnosed HIV-infected individuals in nonmarital partnerships. We defined the PS uptake cascade as a denominator-numerator linkage that used the same group of individuals for all stages of PS. We defined 4 phased indicators for the PS procedure, as follows: number of sexual partners, number of enumerated sexual partners (with names or contact information), number of sexual partners tested for HIV, and HIV-positive sexual partners.

Participants recruited in this study met the following criteria: (1) were infected with HIV through sexual behavior, (2) had been diagnosed with HIV for the first time in 2022 in Zhejiang province and were reported in the national HIV/AIDS information system [33,34], (3) had nonmarital sexual partners, and (4) voluntarily agreed and were able to give informed consent to participate in the study.

### Study Setting and Recruitment Procedures

This study was conducted in Zhejiang province in southeastern China from January 1, 2022, to December 31, 2022. Participants were recruited by staff in all 90 county-level CDCs of Zhejiang province once they were diagnosed with HIV. After participants’ informed consent, epidemiological information was collected in person by CDC personnel. Participants were invited to recall the number and characteristics of nonmarital sexual partners and enumerate their nonmarital sex partners and were offered HIV testing services by trained CDC staff. There are 4 main approaches to HIV testing for sex partners in Zhejiang Province [15]. Participants can opt for any approach, and the test promotion process can be initiated with the participants’ consent by either themselves or health care professionals.

In Zhejiang province, there were 4279 individuals diagnosed with HIV in 2022. Of these, 3960 were transmitted through nonmarital sexual behavior and met the participant inclusion criteria. A total of 3509 participants (3509/3960, 88.6%) were finally included in the study.

### Measures and Definitions of Variables

We describe the PS uptake cascade from the perspective of both the nonmarital sexual partner and the index case using the following measurements: (1) median number of nonmarital sex partners before HIV confirmation, (2) percentage of enumerated nonmarital sexual partners, (3) percentage of HIV testing uptake among enumerated nonmarital sex partners, and (4) the rate of HIV-positive diagnoses among enumerated nonmarital sex partners who were tested for HIV.

By reviewing the previous literature [24,27,28,33] and incorporating the classification of high-risk behavior in the Chinese HIV/AIDS Case Reporting system (Comprehensive Response Information Management System) [34], we defined nonmarital sexual partners of index cases as one the following 5 categories: (1) heterosexual commercial partners, defined as male-female sex relationships in exchange for money; (2) heterosexual noncommercial partners, defined as male-female sex relationships without the exchange of money; (3) homosexual commercial partners, defined as men with whom the male index case had sex in exchange for money; (4) homosexual noncommercial casual partners, defined as men with whom the male index case had occasional sex (eg, one-night stands) or who had a sexual relationship for less than 3 months; (5) homosexual noncommercial regular partners, defined as men with whom the male index case had a sexual relationship for more than 3 months.

We collected and included the demographic and HIV-related characteristics of the participants as covariates to explore the factors associated with PS uptake at the index case level. These variables included gender; age at HIV diagnosis; marital status; ethnicity (defined as Han or minority); household registration (Zhejiang Province or other provinces); education; history of STD; residential area in Zhejiang province (the northern cities of Hangzhou, Jiaxing, and Huzhou; the middle cities of Ningbo, Shaoxing, Jinhua, Quzhou, and Taizhou; and southern cities of Wenzhou, Taizhou, and Lishui); medical route of diagnosis; HIV stage at diagnosis (HIV or AIDS); transmission route (heterosexual or homosexual); and presence of a regular nonmarital sexual partner, defined as a nonmarital sex partner with whom the index case had a sexual relationship for more than 3 months.

### Statistical Analysis

We conducted descriptive analyses. Continuous variables are presented as the median (IQR), and categorical variables are presented as the frequency or proportion. We used chi-square tests in univariate analyses to assess the differences in the distribution of the following primary outcomes of interest across participants with various demographic and sexual behavior characteristics: whether the index case has enumerated any nonmarital sex partners (yes, no), whether the index case has nonmarital sex partners who were tested for HIV (yes, no), and whether the index case has any nonmarital sex partner who was tested for HIV and had a positive result (yes, no). We used binary logistic regression analysis to determine the association between the demographic and HIV-related characteristics of the index case and primary outcomes of interest. Factors in the univariate analyses with a 2-sided *P* value <.10 and factors previously identified to be associated with the 3 outcomes of interest were included in the multivariate model (backward stepwise regression). We calculated the adjusted odds ratio (aOR) and 95% CI. *P*<.05 was considered statistically significant in the logistic regression model. All data cleaning, data processing, and statistical analyses were performed in Microsoft Excel Office 365 and Python 3.7.1.

## Results

### Sociodemographic Characteristics of the Study Participants

Of the 3509 participants, 86.9% (3050/3509) were men, over one-half (1835/3509, 52.3%) were diagnosed at 25 years to 49 years old, and over one-third of the participants were unmarried (1438/3509, 41%) or married (1316/3509, 37.5%). Most of the participants were of Han ethnicity (3329/3509, 94.9%), had household registrations in other provinces (1810/3509, 51.6%), and were diagnosed at the HIV stage (2361/3509, 67.3%), as presented in Table 1.

**Table 1:** Demographic characteristics and univariate analyses of factors associated with enumeration of nonmarital sex partners, HIV testing, and HIV positivity among individuals with newly diagnosed sexually transmitted HIV in Zhejiang Province in 2022.

Participant characteristics		Participants (N=3509), n (%)	Nonmarital sex partners (n=1688)			Nonmarital sex partners who were tested for HIV (n=890)			Nonmarital sex partners who tested HIV positive (n=276)		
			n (%)	*χ*^2^ (*df*)	*P* value	n (%)	*χ*^2^ (*df*)	*P* value	n (%)	*χ*^2^ (*df*)	*P* value
**Gender**				22.942 (1)	<.001		36.841 (1)	<.001		7.475 (1)	.02
	Male	3050 (86.9)	1515 (49.7)			773 (51.0)			227 (29.4)		
	Female	459 (13.1)	173 (37.7)			117 (67.6)			49 (41.9)		
**Age (years)**				82.913 (3)	<.001		95.168 (3)	<.001		86.579 (3)	<.001
	<25	554 (15.8)	320 (57.8)			193 (60.3)			43 (22.3)		
	25-49	1835 (52.3)	942 (51.3)			493 (52.3)			142 (28.8)		
	50-59	674 (19.2)	281 (41.7)			138 (49.1)			60 (43.5)		
	≥60	446 (12.7)	145 (32.5)			66 (45.5)			31 (47.0)		
**Marital status**				84.572 (3)	<.001		90.53 (3)	<.001		75.083 (3)	<.001
	Unmarried	1438 (41)	820 (57)			453 (55.2)			110 (24.3)		
	Married	1316 (37.5)	552 (41.9)			269 (48.7)			102 (37.9)		
	Divorced/widowed	717 (20.4)	308 (43)			165 (53.6)			64 (38.8)		
	Unknown	38 (1.1)	8 (21.1)			3 (37.5)			0 (0)		
**Ethnicity**				2.157 (1)	.14		2.487 (1)	.29		5.982 (1)	.05
	Han	3329 (94.9)	1611 (48.4)			852 (52.9)			258 (30.3)		
	Minority	180 (5.1)	77 (42.8)			38 (49.4)			18 (47.4)		
**Household registration**				1.703 (1)	.19		5.047 (1)	.08		7.744 (1)	.02
	Zhejiang province	1699 (48.4)	798 (47)			402 (50.4)			136 (33.8)		
	Other provinces	1810 (51.6)	890 (49.2)			488 (54.8)			140 (28.7)		
**Education**				62.491 (3)	<.001		64.049 (3)	<.001		40.124 (3)	<.001
	Primary school or illiterate	854 (24.3)	355 (41.6)			181 (51)			67 (37)		
	Junior high school	1144 (32.6)	502 (43.9)			260 (51.8)			92 (35.4)		
	Senior high school	733 (20.9)	371 (50.6)			205 (55.3)			53 (25.9)		
	College or higher	778 (22.2)	460 (59.1)			244 (53)			64 (26.2)		
**Occupation**				28.83 (6)	<.001		36.387 (6)	<.001		41.306 (6)	<.001
	Worker	735 (20.9)	374 (50.9)			185 (49.5)			61 (33)		
	Farmer	826 (23.5)	376 (45.5)			190 (50.5)			69 (36.3)		
	Service industry employee	881 (25.1)	439 (49.8)			237 (54)			63 (26.6)		
	Housework/unemployed	666 (19)	305 (45.8)			175 (57.4)			66 (37.7)		
	Cadres/retired personnel	181 (5.2)	75 (41.4)			39 (52)			7 (17.9)		
	Student	108 (3.1)	73 (67.6)			43 (58.9)			6 (14)		
	Other	112 (3.2)	46 (41.1)			21 (45.7)			4 (19)		
**STD^a^ history**				8.375 (2)	.02		11.599 (2)	.02		3.682 (2)	.45
	Yes	529 (15.1)	276 (52.2)			133 (48.2)			37 (27.8)		
	No	2723 (77.6)	1306 (48)			703 (53.8)			221 (31.4)		
	Unknown	257 (7.3)	106 (41.2)			54 (50.9)			18 (33.3)		
**Resident** **ial area in Zhejiang**				58.091 (2)	<.001		111.499 (2)	<.001		40.434 (2)	<.001
	North	1298 (37)	725 (55.9)			323 (44.6)			84 (26)		
	Middle	1321 (37.6)	609 (46.1)			390 (64)			146 (37.4)		
	South	890 (25.4)	354 (39.8)			177 (50)			46 (26)		
**Medical route for diagnosis**				67.195 (3)	<.001		75.93 (3)	<.001		48.03 (3)	<.001
	VCT^b^	730 (20.8)	414 (56.7)			235 (56.8)			72 (30.6)		
	Hospitals (expected for STD clinic)	1833 (52.2)	778 (42.4)			384 (49.4)			122 (31.8)		
	STD clinic	430 (12.3)	255 (59.3)			132 (51.8)			33 (25)		
	Others	516 (14.7)	241 (46.7)			139 (57.7)			49 (35.3)		
**HIV stage at diagnosis**				16.405 (1)	<.001		22.895 (1)	<.001		26.567 (1)	<.001
	HIV	2361 (67.3)	1192 (50.5)			653 (54.8)			186 (28.5)		
	AIDS	1148 (32.7)	496 (43.2)			237 (47.8)			90 (38)		
**Transmission route**				150.166 (1)	<.001		174.167 (1)	<.001		138.727 (1)	<.001
	Heterosexual	1840 (52.4)	704 (38.3)			321 (45.6)			122 (38)		
	Homosexual	1669 (47.6)	984 (59)			569 (57.8)			154 (27.1)		
**Have a regular nonmarital sexual partner**				218.99 (1)	<.001		261.41 (1)	<.001		224.327 (1)	<.001
	No	2503 (71.3)	1006 (40.2)			470 (46.7)			115 (24.5)		
	Yes	1006 (28.7)	682 (67.8)			420 (61.6)			161 (38.3)		

^a^STD: sexually transmitted disease.

^b^VCT: voluntary counseling and testing.

### PS Uptake Cascade by Sex Partner Category

A total of 14,556 sex partners were disclosed by 3509 participants, with a median of 3 sex partners per participant. In addition, 2507 sex partners with contact information were enumerated by participants, accounting for 17.2% (2507/14,556) of the total number of sex partners. Among the enumerated sex partners, 43.1% (1090/2507) were tested for HIV, and 309 of them tested positive, for a positive rate of 28.3% (309/1090).

**Figure 1:**
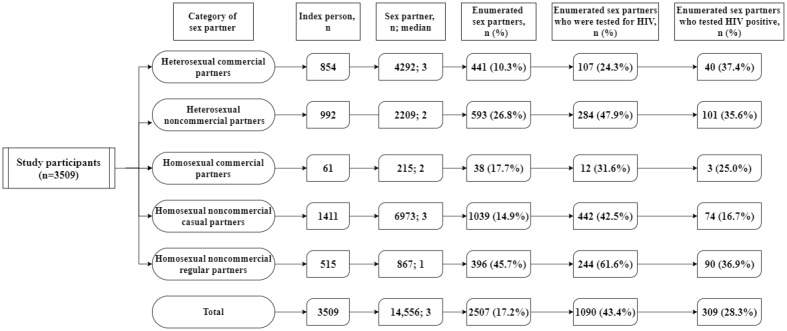
Partner service uptake cascade by sex partner categories among individuals who were newly diagnosed with HIV/AIDS in Zhejiang province in 2022 and had nonmarital sex partners.

The PS uptake cascade by sex partner category is presented in Figure 1. A total of 854 participants recalled having had 4292 heterosexual commercial partners, with a median of 3 partners, the highest median number of sex partners among the 5 groups and the same as homosexual noncommercial casual partners (1411 participants recalled 6973 partners). The percentages of participants who enumerated sexual partners in these 2 categories were the lowest, at 10.3% (441/4292) and 14.9% (1039/6973), respectively.

Heterosexual noncommercial partners (284/593, 47.9%) had the highest percentage of their enumerated sex partners undergo HIV tests, followed by homosexual noncommercial regular partners (442/1039, 42.5%). Among the enumerated sex partners who were tested for HIV, the following categories had HIV-positive rates >30%: heterosexual commercial partners (40/107, 37.4%), homosexual noncommercial regular partners (90/244, 36.9%), heterosexual noncommercial partners (101/284, 35.6%).

### PS Uptake Cascade and Associated Factors at the Participant Level

#### Nonmarital Sex Partner Enumeration

Of the 3509 participants, 48.1% (1688/3509) enumerated their nonmarital sex partners with their names. Table 1 presents the percentage of enumerated sex partners with their names by demographic group. More than one-half of the following participant groups enumerated their sex partners with their names: diagnosed at age <25 years (320/554, 57.8%), who were unmarried (820/1438, 57%), with college or higher education (460/778, 59.1%), resident of northern Zhejiang (725/1298, 55.9%), were diagnosed through voluntary counseling and testing (414/730, 56.7%), HIV transmission through homosexual behavior (984/1669, 59%), regular nonmarital sexual partners (682/1006, 67.8%). There were significant differences in these percentages for the following groups: gender, age, marital status, education, occupation, STD history, residential area in Zhejiang, medical route for diagnosed, HIV stage at diagnosis, transmission route, and the presence of a regular nonmarital sex partner (Table 1). After adjusting for sociodemographic characteristics in the multivariate logistic regression model, gender, age, education, occupation, residential area in Zhejiang, transmission route, and the presence of a regular nonmarital sex partner were associated with the enumeration of sex partners (Table 2).

**Table 2:** Multifactor analysis of factors associated with nonmarital sex partner enumeration, HIV testing, and HIV positivity among individuals newly diagnosed with sexually transmitted HIV in Zhejiang Province in 2022.

Participant characteristics		Number of nonmarital sex partners		Nonmarital sex partners who were tested for HIV		Nonmarital sex partners who tested HIV positive	
		aOR^a^ (95% CI)	*P* value	aOR (95% CI)	*P* value	aOR (95% CI)	*P* value
**Gender**							
	Male (reference)	N/A^b^	N/A	N/A	N/A	—^c^	—
	Female	0.727 (0.572-0.926)	.01	2.700 (1.856-3.928)	.01	—	—
**Age (years)**							
	<25 (reference)	N/A	N/A	—	—	N/A	N/A
	25-49	1.017 (0.811-1.274)	.89	—	—	1.137 (0.735-1.758)	.57
	50-59	0.923 (0.694-1.228)	.58	—	—	2.04 (1.198-3.475)	.009
	≥60	0.609 (0.435-0.853)	.004	—	—	2.379 (1.193-4.743)	.01
**Marital status**							
	Unmarried	—	—	—	—	—	—
	Married	—	—	—	—	—	—
	Divorced/widowed	—	—	—	—	—	—
	Unknown	—	—	—	—	—	—
**Ethnicity**							
	Han	—	—	—	—	—	—
	Minority	—	—	—	—	—	—
**Household registration**							
	Zhejiang province	—	—	—	—	—	—
	Other provinces	—	—	—	—	—	—
**Education**							
	Primary school or illiterate (reference)	N/A	N/A	—	—	—	—
	Junior high school	0.734 (0.597-0.904)	.004	—	—	—	—
	Senior high school	0.776 (0.604-0.995)	.046	—	—	—	—
	College or higher	0.954 (0.728-1.25)	.73	—	—	—	—
**Occupation**							
	Worker (reference)	N/A	N/A	—	—	N/A	N/A
	Farmer	1.206 (0.964-1.507)	.10	—	—	0.835 (0.52-1.343)	.46
	Service industry employee	0.717 (0.576-0.892)	.003	—	—	0.699 (0.453-1.08)	.11
	Housework/unemployed	0.939 (0.746-1.183)	.60	—	—	1.092 (0.693-1.721)	.71
	Cadres/retired personnel	0.673 (0.463-0.977)	.04	—	—	0.374 (0.152-0.922)	.03
	Student	1.183 (0.721-1.939)	.51	—	—	0.409 (0.152-1.100)	.08
	Other	0.681 (0.444-1.045)	.08	—	—	0.452 (0.141-1.453)	.18
**STD^d^ history**							
	Yes (reference)	—	—	N/A	N/A	—	—
	No	—	—	1.426 (1.086-1.873)	.01	—	—
	Unknown	—	—	1.081 (0.672-1.738)	.75	—	—
**Residential area in Zhejiang**							
	North (reference)	N/A	N/A	N/A	N/A	N/A	N/A
	Middle	0.75 (0.635-0.887)	.001	2.47 (1.954-3.123)	<.001	1.487 (1.058-2.088)	.02
	South	0.615 (0.508-0.744)	<.001	1.415 (1.084-1.848)	.01	0.849 (0.545-1.321)	.47
**Medical route for diagnosis**							
	VCT^e^ (reference)	N/A	N/A	—	—	—	—
	Hospitals (expected for STD clinic)	0.845 (0.694-1.030)	.10	—	—	—	—
	STD clinic	1.200 (0.926-1.556)	.17	—	—	—	—
	Others	1.024 (0.798-1.315)	.85	—	—	—	—
**HIV stage at diagnosis**							
	HIV (reference)	N/A	N/A	—	—	N/A	N/A
	AIDS	0.863 (0.74-1.008)	.06	—	—	1.358 (0.973-1.898)	.07
**Transmission route**							
	Heterosexual (reference)	N/A	N/A	N/A	N/A	—	—
	Homosexual	1.829 (1.533-2.181)	<.001	2.378 (1.897-2.981)	<.001	—	—
**Have a regular nonmarital sexual partner**							
	No (reference)	N/A	N/A	N/A	N/A	N/A	N/A
	Yes	3.017 (2.56-3.554)	<.001	1.725 (1.403-2.122)	<.001	1.962 (1.454-2.647)	<.001

^a^aOR: adjusted odds ratio.

^b^N/A: not applicable.

^c^Not included in the model.

^d^STD: sexually transmitted disease.

^e^VCT: voluntary counseling and testing.

#### Nonmarital Sex Partners Who Were Tested for HIV

Among participants who had enumerated their nonmarital sex partners with their names, 52.7% (890/1688) had enumerated sex partner who were tested for HIV. This percentage was higher among participants who were female (117/173, 67.6%), were diagnosed at <25 years old (193/320, 60.3%), resided in the middle of Zhejiang province (390/609, 64%), were infected through homosexual behavior (569/984, 57.8%), and had a regular nonmarital sexual partner (420/682, 61.6%; Table 1).

After adjusting for sociodemographic characteristics in the multivariate logistic regression model, gender, STD history, residential area in Zhejiang, transmission route, and the presence of a regular nonmarital sexual partner were associated with HIV testing uptake (Table 2).

#### HIV-Positive Rate of Nonmarital Sex Partners Tested for HIV

Among the participants with nonmarital sex partners who were tested for HIV, 31% (276/890) had at least 1 nonmarital sex partner who tested positive (Table 1). This percentage was higher among participants who were female (49/117, 41.9%), were diagnosed at 50 years or older (91/204, 44.6%), were married (102/269, 37.9%) or divorced/widowed (64/165, 38.8%), were an ethnic minority (18/38, 47.4%), were farmers (69/190, 36.3%) or houseworkers or unemployed (66/175, 37.7%), resided in the middle of Zhejiang province (146/390, 37.4%), were diagnosed at the AIDS stage (90/237, 38%), and had regular nonmarital sexual partners (161/420, 38.3%).

After adjusting for sociodemographic characteristics in the multivariate logistic regression model, age at HIV diagnosis, occupation, residential area in Zhejiang Province, and the presence of a regular nonmarital sex partner were associated with HIV-positive diagnoses in nonmarital sex partners who were tested for HIV. Compared with participants who were diagnosed at the age of <25 years, resided in northern Zhejiang province, and did not have a regular nonmarital sex partner, those who were diagnosed at the age of 50 years to 59 years (aOR 2.04, 95% CI 1.198-3.475) or ≥60 years (aOR 2.379, 95% CI 1.193-4.743), resided in the middle of Zhejiang province (aOR 1.487, 95% CI 1.058-2.088), and had regular nonmarital sexual partners (aOR 1.962, 95% CI 1.454-2.647) were more likely to have HIV-positive nonmarital sex partners. Compared with workers, participants who were cadres or retired personnel (aOR 0.374, 95% CI 0.152-0.922) were less likely to have HIV-positive nonmarital sex partners (Table 2).

## Discussion

### Principal Findings

Our study described the utilization of PS among HIV-positive individuals with nonmarital sex partners in Zhejiang province, China. Based on this cross-sectional study of 3509 participants, 43.1% of enumerated nonmarital sex partners were tested for HIV, and 28.3% of them tested HIV positive. Compared with the married partners of people diagnosed with HIV in China [21], the testing rate of nonmarital sex partners in this study was lower, and the HIV-positive rates were similar. This underscores the urgent need to improve HIV testing among nonmarital sex partners. Furthermore, with only 17.9% of all nonmarital sex partners being enumerated, a large number of individuals are unaware of their HIV infection risk. This gap is mainly concentrated in heterosexual commercial partners, with only 10.3% of them being enumerated and 24.3% undergoing HIV testing, resulting in the highest HIV-positive rate of 37.4% among the 5 sex partner groups. In addition, results from the multivariate analysis revealed that age was a predictor of nonmarital sex partner enumeration and HIV positivity; compared with those diagnosed at a younger age, those who were diagnosed at 50 years or older were less likely to enumerate their nonmarital sex partners and more likely to have HIV-positive nonmarital partners. Heterosexual commercial sex behavior is the main route of HIV transmission among older men, who are more likely to use condoms inconsistently and have multiple partners, which further leads to transmission within married couples [35-37], especially in rural areas where heterosexual commercial sex behavior occurs in concealed places, such as low-grade venues or rental rooms [38,39]. In addition, the rate of HIV testing among female sex workers and clients is low [40,41] and is associated with the frequency of risk behaviors, HIV infection risk awareness, and behavior intervention. Efforts to locate partners will be an important consideration for PS implementation; however, female sex workers may not consistently collect names and contact information for their clients, so this information may not be readily available for partner notification [42]. Molecular analysis has been used to identify local HIV transmission hot spots with high efficiency [43,44]. Assisted partner notification that incorporates HIV self-testing and outreach by a community health worker from community-based organizations can increase the uptake of HIV testing among key populations [14,20]. Further studies can be performed to identify the key node in HIV transmission networks through molecular analysis and provide them with PS through community health workers among risk groups with heterosexual commercial sex behavior. This can help with timely identification of HIV-positive individuals and provide postexposure prophylaxis for their partners to avoid HIV transmission as early as possible.

From the participant perspective, 48.1% (1688/3509) enumerated their nonmarital sex partners, 52.7% (890/1688) had enumerated nonmarital sex partners who underwent HIV testing, and 31% (276/890) had nonmarital sex partners who tested positive for HIV. Our study revealed that gender and transmission route were associated with both nonmarital sex partner enumeration and HIV testing. Male participants and those who were infected with HIV through heterosexual behavior were less likely to have their nonmarital sex partners tested for HIV. With lower rates of partner enumeration and HIV testing by male participants and those within heterosexual relationships, women in nonmarital relationships are vulnerable and more likely to be diagnosed late. In sub-Saharan Africa, HIV self-testing appears to be a safe and effective strategy among the general population, facilitating linkage to prevention and care, with few reports of harm [45,46]. Health care providers’ skills regarding assisting with PS should be improved, and equal efforts should be made to initiate HIV testing among men with nonmarital heterosexual partners, provide customized services by taking the characteristics of relationships and timeliness of partner notification into consideration [29], prioritize confidentiality, and avoid adverse outcomes, such as violence after HIV testing of female partners [31].

Consistent with prior studies [20,47,48], our study revealed that, compared with participants who only had casual nonmarital partners, those who had regular nonmarital sex partners were more likely to enumerate their nonmarital sex partners, have them tested for HIV, and have HIV-positive nonmarital sex partners. With the increase in seeking anonymous sex partners through social network apps [49], the greatest obstacle to partner notification among MSM is a lack of contact information [50], especially for casual partners [20], and the same situation exists among heterosexual nonmarital relationships, particularly among casual partners [48]. Assisted PS has been shown to be a safe, efficient, effective strategy for increasing the number of sex partners of HIV-positive individuals who undergo HIV testing [16,45]. MSM who had received encouragement from a trained provider to disclose their HIV status to sex partners were more likely to notify both regular and casual partners [23]. Information-assisted partner notification may be an acceptable option to reach sexual partners for whom limited contact information is available [15]. Due to the large number of casual sexual partners, health care providers should prioritize information collection from casual partners and improve PS skills for this population.

Our study shows that the residential area in Zhejiang province was associated with nonmarital sex partner enumeration, HIV testing, and HIV positivity. Compared with participants who resided in northern Zhejiang, those who lived in the middle of the province were less likely to enumerate their nonmarital sex partners and more likely to have nonmarital sex partners who were tested for HIV and were HIV positive. The geographic disparity of PS utilization could be attributed to the transmission route and age difference in the HIV epidemic in different areas or health care resources [51], with homosexual transmission concentrated in the northern region of Zhejiang, symbolized by the city of Hangzhou [52], and heterosexual transmission and older adults predominating in the central and southern regions.

There were several limitations in our study. First, because information on sexual partners was mostly recalled by the participants, recall bias is inevitable. Additionally, owing to social desirability bias and concerns about stigma, participants could have hesitated to disclose information about their sexual partners and further introduce partner testing services. Second, we investigated a limited set of variables associated with PS only from the perspective of the participants, and some sociopsychological predictors like fear of exposure, discrimination, and economic factors as well as inter-partner features, such as condom usage and communication and social interaction patterns, may need to be explored in future research to uncover significant predictors of PS uptake and lead to more comprehensive intervention strategies. Third, it takes time for health care professionals to build trust and successfully implement PS for the index case; nevertheless, because this was a cross-sectional study, the proportion of PS uptake may be underestimated, particularly for index cases diagnosed in the later months of 2022.

### Conclusions

A significant gap in PS utilization was observed among HIV-positive individuals with nonmarital sex partners in eastern China, especially among heterosexual commercial partnerships. Gender, age, transmission category, and the presence of a regular nonmarital partner were predictors of partner enumeration or testing in nonmarital relationships. Our analysis also revealed a higher HIV positivity rate among nonmarital sexual partners of participants who were diagnosed when they were older than 50 years and had regular nonmarital partners. Our findings highlight the critical need to boost the overall utilization of PS among HIV-positive individuals older than 50 years and those with heterosexual nonmarital sex partners. These findings will contribute to the understanding of PS among HIV-positive individuals with nonmarital sex partners and develop a pilot study to optimize the PS strategy for this group.

## Abbreviations

aOR: adjusted odds ratio
CDC: Center for Disease Control and Prevention
MSM: men who have sex with men
OR: odds ratio
PS: partner service
STD: sexually transmitted disease
UNAIDS: Joint United Nations Programme on HIV/AIDS
WHO: World Health Organization

## Data Availability

The data analyzed in our study are available from the corresponding author on reasonable request.
